# A retrospective study on the impact of comorbid depression or anxiety on healthcare resource use and costs among diabetic neuropathy patients

**DOI:** 10.1186/1472-6963-9-111

**Published:** 2009-06-30

**Authors:** Luke Boulanger, Yang Zhao, Yanjun Bao, Mason W Russell

**Affiliations:** 1 Health Economic Research and Quality of Life Evaluation, Abt Bio-Pharma Solutions, Inc, Lexington, MA, USA; 2 Global Health Outcomes, Eli Lilly and Company, Inc, Indianapolis, IN, USA

## Abstract

**Background:**

Diabetic neuropathy (DN) is a common complication of diabetes that has significant economic burden, especially for patients with comorbid depression or anxiety. This study examines and quantifies factors associated with healthcare costs among patients diagnosed with diabetic neuropathy (DN) with or without a comorbid diagnosis of depression or anxiety (DA) using retrospective administrative claims data. No study has examined the differences in economic outcomes depending on the presence of comorbid DA disorders.

**Methods:**

Over-age-18 individuals with 1+ diagnosis of DN in 2005 were selected. The first observed DN claim was considered the "index date." All individuals had a 12-month pre-index and follow-up period. For both under-age-65 commercially insured and over-age-65 individuals with employer-sponsored Medicare supplemental insurance, we constructed 2 subgroups for individuals with DA (DN-DA) or without (DN-only). Patients' clinical characteristics over pre-index period were compared. Multivariate regressions were performed to assess whether DN-DA patients had higher utilization of healthcare resources and costs than DN-only patients, controlling for demographic and clinical characteristics.

**Results:**

We identified 16,831 DN-only and 1,699 DN-DA patients in the Medicare supplemental cohort, as well as 17,205 and 3,105 in the commercially insured. DN-DA patients had higher prevalence of diabetes-related comorbidities for cardiovascular disease, cerebrovascular/peripheral vascular disease, nephropathy, obesity, and hypoglycemic events than DN-only patients (all p < 0.05). Controlling for differences in demographic and clinical characteristics, DN-DA patients had $9,235 (p < 0.05) higher total healthcare costs than patients with DN-only among those with Medicare supplemental coverage ($26,718 vs. $17,483), and $10,389 (p < 0.05) more total costs among commercially insured ($29,775 vs. $19,386). Factors associated with increased costs included insurance type, geographical region, diabetes-related comorbidities, and insulin therapy.

**Conclusion:**

These findings indicate that the healthcare costs were significantly higher for DN patients with depression or anxiety relative to those without such comorbid disorders.

## Background

Diabetes mellitus is a chronic condition that has been estimated to affect over 20 million people or 7% of the total population in the U.S. in 2005 [1]. Diabetic neuropathy (DN) is a common complication of diabetes, in which nerves are damaged as a result of hyperglycemia. Painful DN manifests itself as an electric, burning, or shooting sensation; however patients with DN can also be asymptomatic. Risk factors for DN include hyperglycemia, hyperlipidemia, high blood pressure, obesity, age of at least 40 years, and having diabetes for at least 25 years [2-4].

The economic burden of symptomatic and non-symptomatic DN is significant. The total annual direct costs of DN and its complications in the U.S. were $4.6–13.7 billion in 2001 [5]. Patients with DN had significantly higher total medical costs than individuals with diabetes but no DN [6]. It has been shown that more than one quarter of total direct costs for diabetes in the U.S. can be attributed to DN [5]. The indirect costs of DN, including missed days of work and decreased productivity, are a considerable component of total costs [7]. Recent research suggests that DN symptoms cause workers to lose approximately $3.65 billion per year in health-related lost productivity [7].

Compared to the general U.S. population, diabetic patients are twice as likely to be diagnosed with depression [8]. Prior studies have estimated that 28% of DN patients have depression and 35% have moderate to severe anxiety [9,10], and a more recent multi-center study reported that 59% of DN patients had either condition [11]. The healthcare resource utilization and associated treatment costs for patients with diabetes comorbid with DN or depression are much higher than those without [6]. However, little has been done to examine resource utilization and costs among DN patients, and no study has examined the differences in these outcomes depending on the presence of comorbid depression or anxiety disorders.

The aim of this study is to fill this gap by employing a retrospective cohort design using a large US administrative claims database to examine and quantify the economic impact of comorbid depression or anxiety disorders on healthcare resource utilization and associated costs for DN patients. Findings from this study can be incorporated into economic models of diabetes care and are relevant to inform healthcare providers of diabetic patients and policy makers interested in the economic impact of DN. Patients who were covered by commercial insurance (18–64 years of age) and patients over age 65 with employer-sponsored Medicare supplemental insurance were assessed separately, and trends were compared across the two populations. Multivariate regression analysis was undertaken to examine the effects of having comorbid depression or anxiety disorders on the likelihood and number of healthcare services, as well as on costs after controlling for differences in demographic and clinical characteristics.

## Methods

### Data Source

The Thomson Medstat MarketScan Commercial Claims and Encounters and Medicare Supplemental databases (2004–2006) were used for the analysis for patients aged 18–64 and 65+, respectively. This database contains administrative claims and eligibility records for approximately 20 million patients in distinct sets of files for commercially insured (i.e., working age adults and their dependants) and Medicare supplemental insured individuals from approximately 45 large health plans geographically located in the United States. The databases include separate files for enrollment records, medical and pharmacy claims. The files are linkable based on an encrypted patient identification number. Enrollment records contain demographic information, including age, sex, and geographic region. Medical claims files include inpatient, outpatient, facility and services claims records. They report up to 15 International Classification of Diseases, 9th Revision, Clinical Modification (ICD-9-CM) codes for diagnoses and up to fifteen ICD-9-CM procedures codes, date of service, place of service, provider type, and plan and patient paid amounts. Pharmacy claims files provide information for each prescription with the National Drug Code (NDC), dispense dates, quantity and days supplied, and plan and patient paid amounts. Because data used in this study were purchased from a third party which had removed identifying information prior to its release, institutional review board (IRB) and similar approvals were neither needed nor sought.

### Identification of Study Population

Medical problems that each individual encountered were identified based on ICD-9-CM codes associated with medical claims. A patient diagnosed with diabetes (ICD-9-CM: 250.xx) was included in the study if the patient had at least one DN claim (250.6x and/or 357.2) [12-14] in the calendar year of 2005, and the first observed DN claim was set as the index date. Patients may or may not have a DN claim in the 12 months prior to the index date. Furthermore, included patients were required to be at least 18 years of age as of the index date and to have continuous health plan enrollment from 12 months prior to the index date (i.e. pre-index period) through 12 months following the index date (i.e. follow-up period).

We separately assessed study patients who were covered by commercial insurance (working age adults 18–64 years of age) and those of age 65 and above with employer-sponsored Medicare supplemental insurance. For each analysis, two study cohorts were identified based on the presence of 1+ medical claim for depression (ICD-9-CM: 296.2, 296.3, 300.4, 309.1, 311.0) or for anxiety disorders (ICD-9-CM: 300.0x, 300.2x, 300.3, 309.81) either in the pre-index period or in the post-index period. DN patients with depression and/or anxiety made up the "DN-DA" cohort and those without such disorders constituted the "DN-only" cohort.

### Study Measures

Patient demographic characteristics in the pre-index period included age, sex, and geographic regions (i.e. northeast, north central, west, south). Dichotomous variables (1 = Yes, 0 = No) were created to measure clinical characteristics over the 12 month pre-index period that included: 1) use of insulin only, use of oral anti-diabetic drugs (OADs) only, or use of insulin and OADs identified based on the NDC codes in pharmacy claims; 2) the presence of diabetes-related co-morbidities and complications identified based on ICD-9-CM codes in medical claims such as:

• Cardiovascular disease (CVD; ICD-9-CM: 390-398.xx, 401.x-403.xx, 404.1, 404.9, 405.xx, 410.xx-417.x, 420.xx-429.xx),

• Cerebrovascular/peripheral vascular disease (CPVD; ICD-9-CM: 430-437.x, 440.xx-444.xx, 447.x-454.x, 457.x-459.xx, 785.4),

• Diabetes related infections (ICD-9-CM: 038.xx, 790.7),

• Other metabolic diseases (ICD-9-CM: 251.3, 270.3, 276.xx),

• Nephropathy (ICD-9-CM: 580.9, 581.81, 581.9, 582.9, 583.xx, 588.8x, 593.9),

• Obesity (ICD-9-CM: 278.xx),

• Retinopathy (ICD-9-CM: 362.0x-362.2x, 362.41, 363.31, 365.44, 366.41),

• Hypoglycemic events (ICD-9-CM: 250.8x, 251.0–251.2),

• Skin problems (ICD-9-CM: 680.x-686.xx, 707.xx), and

• Leg amputation (ICD-9-CM: 278.80–278.82, 278.84, 278.86);

and 3) being hospitalized. Overall and diabetes-related healthcare expenditures (in 2006 dollars) in the pre-index period were also calculated. Diabetes-related expenditures were estimated based on the medical service claims with diabetes diagnoses (ICD-9-CM: 250.xx) coded anywhere in the diagnosis file and medication costs recorded in the pharmacy claims. Diabetes-related medication costs were estimated based on prescription of insulin and OADs.

All-cause healthcare resource utilization and associated direct costs in the follow-up period were quantified for major service components. Specifically, the percentage of patients with any medical services and the number of services for each patient were calculated for components of healthcare resource utilization including hospitalization, skilled nursing facilities (SNF), emergency room (ER), hospital outpatient, home health, and outpatient physician office visits. Similarly, DN-related healthcare utilization and costs were extracted based on the medical service claims with such diagnoses coded anywhere in the diagnosis file. DN-related medication costs were estimated based on prescriptions of pharmacologic therapies recommended for DN treatment [15]: 1) tricyclic antidepressants, 2) venlafaxine, 3) duloxetine, 4) pregabalin, 5) gabapentin, and 6) opioids (e.g., tramadol, oxycodone, morphine, hydrocodone, methadone, levorphanol). All costs included deductibles, copayments, coinsurance, and coordination-of-benefits payments recorded in the databases, and all costs were adjusted to 2006 U.S. dollars using the medical component of the consumer price index.

### Analysis

Descriptive statistics were summarized. Percentages were reported for categorical variables and cohort differences were analyzed using Cochran-Mantel-Haenszel tests. For continuous variables, mean and standard deviations were reported and student t-tests were used to analyze cohort differences. Logistic regressions were employed to analyze effects of comorbid depression or anxiety disorders on the likelihood of resource use. Ordinary least squares (OLS) regressions were used to assess the association between comorbid disorders and the number of services, whereas generalized least squares models (assuming a gamma distribution specification) were employed for healthcare costs. We conducted our analyses for commercially-insured and Medicare-insured populations separately because the reimbursed amount by Medicare is generally lower than the commercial insurance companies. Explanatory variables in all regressions included age groups, male gender, insurance type, geographic regions, each of the diabetes-related complications and comorbidities listed above, and pre-index use of insulin or OADs. All analyses were conducted using SAS version 9.1 (SAS Institute, Inc., Cary, North Carolina), and findings of p values of < 0.05 were considered statistically significant.

## Results

### Sample Size

**Figure 1:**
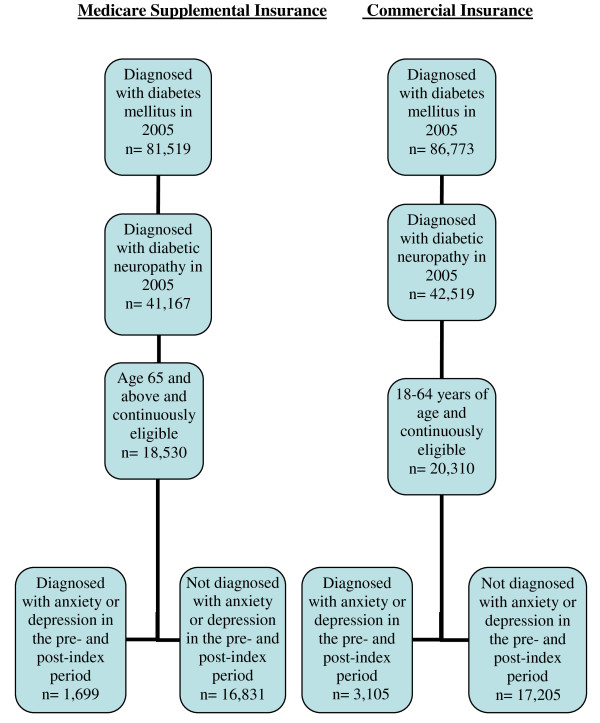
**Sample size of study population**.

Figure 1 illustrates how sample size was attained for both commercially insured and Medicare supplemental covered patients. Patients diagnosed with diabetes mellitus and diabetic neuropathy in 2005 were included in the study. Commercial patients age 18–64 years and Medicare patients 65 years and older were required to be continuously eligible from the 12 months prior through the 12 months after the index date. Furthermore, patients were classified based on whether or not they were diagnosed with depression or anxiety in the 12 months pre- or post-index period.

### Prevalence Estimates

About 50% of all patients diagnosed with diabetes in 2005 also had at least 1 DN diagnosis (Figure 1), which is similar to the rate summarized in a recent review [16]. The DN prevalence rate was slightly higher among patients with Medicare supplemental insurance than those with commercial coverage. Of all DN patients identified in 2005, approximately 4% were diagnosed with depression and/or anxiety disorders, and the prevalence rate was higher for females (5.1%) than for males (3.4%) (data not shown). About 3.1% of DN patients with Medicare supplemental insurance had comorbid depression or anxiety disorders compared with 5.4% among patients with commercial coverage (data not shown).

### Demographic Characteristics

The study identified a total of 18,530 DN patients of age 65 and above with Medicare supplemental insurance, of whom 1,699 (9%) were in the DN-DA cohort (Table 1). Among those in the DN-DA cohort, 67.9% with depression only, 21.7% were diagnosed with anxiety only, and 10.4% had both depression and anxiety. Of the 20,310 commercially insured working age patients between 18–64 years of age, 3,105 (15%) were in the DN-DA cohort. The majority of patients in the DN-DA cohort (68.3%) were diagnosed with depression only, 18.3% were diagnosed with anxiety only, and 13.4% had both depression and anxiety. The mean age was similar between DN-only and DN-DA patients (76 vs. 75 years, p = 0.44) among those with Medicare supplemental insurance, however, DN-DA patients among the commercially insured were significantly younger than DN-only patients (54 vs. 55 years, p < 0.05). The DN-DA cohort of both patient populations had a significantly lower proportion of male patients (44% vs. 53% for those with Medicare supplemental insurance, 41% vs. 54% for the commercial insured, both p < 0.05) than the DN-only cohort. The majority of Medicare supplemental insured patients were enrolled in health plans with comprehensive coverage, while the majority of commercially insured patients had coverage through preferred provider organizations (PPO).

**Table 1:** Pre-index demographic characteristics by insurance type and cohort

	**Medicare supplemental insurance**			**Commercial insurance**		
	
	**DN-only**	**DN-DA**		**DN-only**	**DN-DA**	
				
**# Observations**	16,831	1,699	p-value*	17,205	3,105	p-value*
**Age (mean, SD)**	75.6 (6.1)	75.4 (6.3)	0.44	55.0 (7.4)	53.7 (7.7)	< 0.05

**Male (%)**	52.9	44.2	< 0.05	53.7	41.1	< 0.05

Plan types (%)**			< 0.05			0.54

Comprehensive	65.2	60.4		16.1	17.3	

PPO	21.6	21.9		47.3	45.8	

HMO	11.8	16.5		20.3	22.8	

Other	0.3	0.1		15.6	13.3	

Missing	1.1	1.0		0.8	0.9	

**Region (%)**			< 0.05			0.19

Northeast	10.8	8.5		8.1	8.0	

North central	41.3	42.1		29.9	31.4	

South	30.2	28.4		43.2	38.6	

West	16.7	20.1		18.0	21.1	

Missing	1.0	0.9		0.7	0.9	

Source: Thomson Medstat MarketScan database for patients with Medicare supplemental and commercial insurance (2004–2005)

* Student t-tests for continuous variables and Cochran-Mantel-Haenszel tests for categorical variables were performed.

** HMO = Health Maintenance Organization; PPO = Preferred Provider Organization

### Clinical Characteristics

The top 3 most prevalent comorbidities were CVD, CPVD, and skin problems for both cohorts across populations (Table 2). The DN-DA patients had significantly higher prevalence rate for all diabetes-related complications and comorbidities (all p < 0.05) except retinopathy than the DN-only patients in both populations. Compared with commercially insured DN patients, those with Medicare supplemental insurance had higher prevalence rate for CVD, CPVD, other metabolic disease, nephropathy, skin problems, and infections related to diabetes.

**Table 2:** pre-index clinical characteristics by insurance type and cohort

	**Medicare supplemental insurance**			**Commercial insurance**		
	
	**DN-only**	**DN-DA**	p-value*	**DN-only**	**DN-DA**	p-value*
				
**# Observations**	16,831	1,699		17,205	3,105	
**Diabetes related complications and comorbidities (%)**						

Cardiovascular disease (CVD)	73.2	81.0	< 0.05	58.3	64.3	< 0.05

Cerebro/peripheral vascular disease (CPVD)	34.8	44.4	< 0.05	18.6	25.0	< 0.05

Other metabolic diseases	9.0	19.2	< 0.05	8.5	16.9	< 0.05

Nephropathy	7.5	10.4	< 0.05	7.0	8.6	< 0.05

Retinopathy	13.8	13.7	0.94	16.0	15.1	0.20

Hypoglycemic events	8.9	12.6	< 0.05	9.9	14.1	< 0.05

Skin problems	21.4	25.3	< 0.05	20.7	23.4	< 0.05

Infections related to diabetes	1.9	3.7	< 0.05	1.4	3.1	< 0.05

Obesity	1.3	3.1	< 0.05	3.8	9.2	< 0.05

Leg amputation	0.2	0.3	0.29	0.1	0.4	< 0.05

**Anti-diabetic treatment (%)**						

No treatment	14.3	15.2	0.28	14.2	16.1	< 0.05

Insulin only	15.1	18.0	< 0.05	18.5	21.8	< 0.05

Oral antidiabetic agent only	49.5	44.6	< 0.05	41.2	33.4	< 0.05

Insulin with oral anti-diabetic therapy	21.1	22.1	0.34	26.2	28.7	< 0.05

**Resource use and costs**						

% Patients with any hospitalization	28.0	46.0	< 0.05	20.1	35.7	< 0.05

Total diabetes-related healthcare expenditures^$ ^(mean, SD)	$3,823 ($11,783)	$7,384 ($21,727)	< 0.05	$6,102 ($18,252)	$10,580 ($29,887)	< 0.05

Total healthcare expenditures^$ ^(mean, SD)	$15,332 ($21,915)	$25,514 ($38,869)	< 0.05	$17,848 ($36,220)	$30,352 ($52,812)	< 0.05

Source: Thomson Medstat MarketScan database for patients with Medicare supplemental and commercial insurance (2004–2005)

* Student t-tests for continuous variables and Cochran-Mantel-Haenszel tests for categorical variables were performed.

$ Expenditures were adjusted to 2006 dollars using the medical component of the consumer price index. SD = standard deviation.

Compared with the DN-only cohort, a significantly higher proportion of DN-DA patients were prescribed insulin for both the Medicare supplemental insured (40% vs. 36%, p < 0.05) and the commercially insured (50% vs. 45%, p < 0.05). The percentage of patients with any inpatient admission was significantly higher for the DN-DA cohort compared to the DN-only cohort for both populations (46% vs. 28% among Medicare supplemental insured, 36% vs. 20% among commercially insured, both p < 0.05). The overall and diabetes-related healthcare expenditures for the Medicare supplemental insured patients were approximately $10,000 and $3,500 higher (both p < 0.05), respectively, for the DN-DA cohort. For commercially insured patients, the difference in the overall healthcare expenditures of DN-DA cohort over DN-only cohort was approximately $12,000 (p < 0.05), while the difference in diabetes-related healthcare expenditures was about $4,500 (p < 0.05).

### Resource Utilization

A significantly higher percentage of DN-DA patients over the follow-up period had inpatient hospital, SNF, ER, and hospital outpatient encounters than DN-only patients in both populations, and DN-DA patients also had longer hospital days, more SNF admissions, and more outpatient physician office visits (Table 3). After controlling for demographic and clinical characteristics in pre-index period, patients with DN-DA were twice as likely to be hospitalized and to have an ER visit as the DN-only patients, and incurred 3 additional hospital days (both p < 0.05).

**Table 3:** Resource use in post-index 12 months by insurance type and cohort

	**Medicare supplemental insurance**			**Commercial insurance**		
	**DN-only**	**DN-DA**	**Odds ratio** **(95% confidence interval) or adjusted mean difference** **(p-value)**	**DN-only**	**DN-DA**	**Odds ratio** **(95% confidence interval) or adjusted mean difference** **(p-value)**

**# Observations**	16,831	1,699		17,205	3,105	

**Inpatient hospital admissions**						

% patients with any admission	34.8%	54.3%	2.0 (1.8, 2.2)^1^	25.6%	43.7%	2.0 (1.8, 2.2)^1^

Number of hospitalized days (mean, SD)	2.6 (8.0)	6.0 (13.4)	2.8 (<0.05)^2^	2.5 (9.1)	5.8 (14.5)	2.5 (<0.05)^2^

**SNF admissions**						

% patients with any admission	9.5%	25.3%	2.9 (2.6, 3.3)^1^	1.9%	4.7%	2.0 (1.6, 2.5)^1^

Number of admissions (mean, SD)	0.8 (3.9)	3.2 (8.8)	2.1 (<0.05)^2^	0.1 (1.5)	0.4 (3.0)	0.04 (<0.05)^2^

**% patients with any ER visit**	21.7%	38.8%	2.1 (1.9, 2.3)^1^	22.8%	37.6%	1.8 (1.6, 2.0)^1^

**% patients with any hospital outpatient visit**	78.1%	81.9%	1.3 (1.1, 1.5)^1^	75.0%	82.4%	1.5 (1.3, 1.6)^1^

**Outpatient physician office visits**						

% patients with any visit	98.8%	98.1%	0.7 (0.4, 0.95)^1^	99.3%	99.6%	1.6 (0.9, 2.9)^1^

Number of visits (mean, SD)	18.5 (12.9)	21.6 (15.1)	2.5 (<0.05)^2^	16.7 (13.1)	24.9 (17.6)	7.1 (<0.05)^2^

**Home health care visits**						

% patients with any visit	52.5%	52.4%	1.0 (0.9, 1.1)^1^	36.5%	45.7%	1.3 (1.2, 1.4)^1^

Number of visits (mean, SD)	2.2 (4.8)	2.6 (5.5)	0.2 (0.06)^2^	2.1 (7.1)	3.2 (8.6)	0.6 (<0.05)^2^

Source: Thomson Medstat MarketScan database for patients with Medicare supplemental and commercial insurance (2004–2005)

^1^Adjusted odds ratio and 95% confidence interval estimated from logistic regression.

^2^Adjusted mean difference and p-value estimated from ordinary least squares regression.

Compared with the commercially insured, a higher proportion of DN-DA (54% vs. 44%) and DN-only patients (35% vs. 26%) with Medicare supplemental insurance were hospitalized (Table 3). For both the DN-only and DN-DA cohorts, the population with Medicare supplemental insurance also had higher utilization rates of SNF admissions as well as home health care visits. Emergency room, hospital outpatient, and physician office encounters were similar between both populations.

### Healthcare Costs

Patients in the DN-DA cohort had higher raw total cost and all its components than those in the DN-only cohort for both populations (Table 4). After adjusting for demographic and clinical characteristics in pre-index period, DN-DA patients with Medicare supplemental insurance had $9,235 (p < 0.05) significantly higher total costs compared with those with DN-only ($26,718 vs. $17,483, data not shown), with the most cost differences from inpatient admission ($4,781, p < 0.05) followed by SNF ($1,654, p < 0.05), outpatient prescription ($718, p < 0.05) and hospital outpatient services ($607, p < 0.05). Among the commercially insured, DN-DA patients incurred $10,389 (p < 0.05) significantly higher adjusted total costs compared to the DN-only patients ($29,775 vs. $19,386 data not shown), with the biggest cost differences from inpatient hospitalizations ($5,749, p < 0.05), hospital outpatient ($1,371, p < 0.05), pharmacy services ($1,361, p < 0.05), and outpatient physician office visits ($977, p < 0.05). For both populations, DN-DA patients had significantly higher DN-related healthcare costs for all components than DN-only patients controlling for differences in demographics and clinical characteristics. More than 85% of the difference in adjusted total DN-related healthcare costs between patients in DN-DA and DN-only cohorts was from inpatient service for both Medicare supplemental and commercial insured, followed by pharmacy expenditures contributing additional 11% or 12%, respectively.

**Table 4:** Healthcare costs in post-index 12 months by insurance type and cohort

	**Medicare supplemental insurance**			**Commercial insurance**		
	DN-only*	DN-DA*	**Adjusted cost difference^1^** **(p-value)**	DN-only*	DN-DA*	**Adjusted cost difference^1^** **(p-value)**

Overall healthcare expenditures (mean, SD):						

Inpatient hospital	$5,405 ($18,093)	$11,544 ($34,481)	$4,781 **	$7,864 (30,569)	$15,994 ($38,452)	$5,749**

Skilled nursing facility	$661 ($4,040)	$2,581 ($8,326)	$1,654 **	$181 ($2,471)	$427 ($2,994)	$171 **

Emergency room	$89 ($452)	$229 ($797)	$91 **	$234 ($1,037)	$564 ($1,967)	$241 **

Hospital outpatient	$2,606 ($9,397)	$3,550 ($7,589)	$607 **	$4,169 ($12,507)	$6,323 ($18,333)	$1,371 **

Outpatient physician office	$2,959 ($4,556)	$3,435 ($3,921)	$284 **	$2,815 ($4,930)	$4,083 ($6,192)	$977 **

Home health care	$532 ($2,797)	$614 ($2,063)	$15	$671 ($3,884)	$1,133 ($6,312)	$235 **

Prescription medications	$4,286 ($3,913)	$5,185 ($4,708)	$718 **	$4,257 ($6,538)	$6,001 ($6,538)	$1,361 **

Total	$18,072 ($26,660)	$30,410 ($46,083)	$9,235 **	$22,437 ($44,179)	$38,200 ($56,718)	$10,389 **

DN-related healthcare expenditures (mean, SD):						

Inpatient	$1,165 ($7,544)	$2,947 ($12,387)	$1,365 **	$2,461 ($12,422)	$5,518 ($19,000)	$2,084 **

Outpatient	$420 ($1,508)	$592 ($2,250)	$91 **	$616 ($2,321)	$706 ($2,282)	$31

Pharmacy	$189 ($549)	$364 ($743)	$172 **	$275 ($994)	$666 ($1,457)	$294 **

Total	$1,773 ($7,749)	$3,904 ($12,864)	$1,594 **	$3,353 ($12,821)	$6,890 ($19,442)	$2,432 **

Source: Thomson Medstat MarketScan database for patients with Medicare supplemental and commercial insurance (2004–2005)

* Mean and standard deviation (in parentheses) are reported for each healthcare service. Expenditures were adjusted to 2006 dollars using the medical services component of the consumer price index.

^1^Adjusted difference estimated from generalized least squares regression with a gamma distribution specification.

** Denotes p < 0.05.

## Discussion

Using the administrative claims from the Thomson MedStat MarketScan databases, this study was the first to examine the impact of comorbid depression and anxiety disorders on healthcare resource use and costs among DN patients separately for the Medicare supplemental insured patients and the commercially insured. Not only were DN patients with depression or anxiety disorders more likely to have encounters for inpatient hospitalization, ER, SNF, and outpatient hospital services than those without such disorders, they also had longer hospital days, more SNF admissions, and more ER and outpatient hospital visits. While the likelihood of using outpatient physician office services was not different between DN-DA and DN-only cohorts, the former was associated with a significantly higher number of the services over the one year follow-up period. Controlling for differences in demographic and clinical characteristics, patients in the DN-DA cohort had significantly higher total costs and higher costs for each of the components than those in the DN-only cohort. For both cohorts in each population, inpatient hospital costs constituted the largest proportion of overall costs. Outpatient prescriptions and hospital outpatient costs were ranked as the second and third highest cost items for the Medicare supplemental insured DN patients; however, they contributed to similar proportion of the total costs for the commercially insured sample.

For both the commercially insured and the Medicare supplemental insured DN patients, the prevalence rates of all other diabetes-related complications and comorbidities except retinopathy were higher among those in the DN-DA cohort than those in the DA-only cohort. Specifically, the biggest differences in prevalence rates between those cohorts were in CVD, CPVD, and other metabolic diseases. Although it is unclear about the causality between depression/anxiety and other diabetes-related complications and comorbidities among DN patients, special attention is needed to monitor other diabetes-related complications and/or comorbidities when managing DN patients comorbid with depression and/or anxiety.

This study found lower utilization rates of many components of healthcare services among the commercially insured patients compared with those with Medicare supplemental insurance. This is especially true for hospital and SNF admissions, as well as home health care visits. However, healthcare costs were higher among the commercially insured patients. One possible explanation for this finding is that the various resource-based payment systems currently employed by Medicare have made the unit cost per healthcare service less expensive than similar services covered by commercial insurance. Despite of the difference between various payment systems, DN patients with depression or anxiety disorders had over $9,000 more total costs than those without such disorders in both populations after controlling for differences in patient demographic and clinical characteristics.

The actual direct costs associated with DN treatment could be much higher than what was captured in the study since DN is under-reported to physicians and subsequently under-treated [17,18]. Older DN patients could be less likely to seek treatment for depression or anxiety disorders than younger patients. Previous research has also found a high rate of comorbid anxiety disorders among patients with depression [19]. Several intervention trials have found that proper treatment for comorbid depression among diabetes patients was associated with lower medical costs [20-22]. Therefore, it is important to recognize that the need for treatment of comorbid depression or anxiety disorders is considerable, particularly among patients with Medicare supplemental insurance, who were also associated with significantly higher levels of resource use compared to the commercially insured patients.

Several limitations need to be noted when interpreting results for this study. Firstly, a selection bias could exist where DN patients with comorbid depression or anxiety disorders had unobserved heterogeneity that correlated with levels of disease severities and study outcomes. Despite the best efforts to capture patient heterogeneity by demographic and clinical characteristics over pre-index period, the study was unable to control for unobserved confounding characteristics using a retrospective study design. Secondly, as patients' actual medical records could not be accessed for the study, the identification of DN, depression or anxiety disorders and other diabetes related complications and comorbidities were based on the ICD-9-CM diagnosis codes in medical claims. This information could be subject to clerical errors or other reporting errors, leading to potentially inaccurate identification of patients' conditions. Additionally, we only required 1 DN diagnosis to identify the DN cohort. We might misclassify some of non-DN patients to the DN cohort due to coding errors. Similarly, the study was unable to directly measure the severity of DN or depression or anxiety disorders, which was found to be a significant predictor of healthcare utilization and costs [23] and to be associated with decreased quality of life outcomes [24]. Thirdly, our study did not assess other pain related conditions between the study cohorts. However, we are not aware of any published studies that report differences in pain-related conditions within subgroups of DN patients. Fourthly, the study calculated direct treatment costs based on plan payment and patient co-payment for each service, and therefore did not capture other indirect costs incurred by patients such as lost productivity and opportunity costs associated with time lost seeking treatment. Also, as over-the-counter medications were not captured in the administrative claims databases, the study could be underestimating the pharmacy utilization and thus associated costs. Furthermore, we may overestimate the diabetes- and DN-related costs as we used diabetes or DN diagnoses recorded anywhere in the claims to assign such costs. We also lack of information regarding the health plan-specific benefit structure, which may influence healthcare utilization. Finally, the results presented here could only be taken as associations instead of causations. While the study found that DN patients with comorbid depression or anxiety disorders used more resources and incurred higher costs for treatment, it cannot be established whether such increased resource use and costs were a result of comorbid disorders.

## Conclusion

This study assessed healthcare resource use and costs between patients diagnosed with DN with and without diagnosed depression or anxiety disorders among those aged 18–64 commercially insured and those of age 65 and above with employer-sponsored Medicare supplemental insurance. We found significantly higher healthcare resource utilization and costs among DN patients who had comorbid depression or anxiety disorders versus those without such diagnoses, even after controlling for demographic and clinical differences. Properly treating these comorbid depression or anxiety disorders among DN patients may have an impact on the economic burden of DN.

## Abbreviations

DN: diabetic neuropathy; DA: depression or anxiety; ICD-9-CM: International Classification of Diseases, 9th Revision, Clinical Modification; OAD: oral anti-diabetic drugs; CVD: Cardiovascular disease; CPVD: Cerebrovascular/peripheral vascular disease; SNF: skilled nursing facilities; ER: emergency room; OLS: ordinary least squares.

## Competing interests

YZ is employed by Eli Lilly and Company, and owns company stocks.

## Authors' contributions

LB made substantial contributions to conception and design, acquisition of data, analysis and interpretation of data, and revising the manuscript for important intellectual content. YZ participated in identifying the study design, critically revising the manuscript, and providing final approval for publication. YB was involved in the analysis and interpretation of data. MR oversaw the development of the study design. All authors read and approved the final manuscript.

## Pre-publication history

The pre-publication history for this paper can be accessed here:


